# A Splice Mutation and mRNA Decay of EXT2 Provoke Hereditary Multiple Exostoses

**DOI:** 10.1371/journal.pone.0094848

**Published:** 2014-04-11

**Authors:** Chen Tian, Rengna Yan, Shuzhen Wen, Xueling Li, Tianfeng Li, Zhenming Cai, Xinxiu Li, Hong Du, Huimei Chen

**Affiliations:** 1 Department of Medical Genetics, Nanjing University School of Medicine, Nanjing, China; 2 Jiangsu Key Laboratory of Molecular Medicine, Nanjing, China; 3 Jinling Hospital, Nanjing University School of Medicine, Nanjing, China; University of Sheffield, United Kingdom

## Abstract

**Background:**

Hereditary multiple exostoses (HME) is an autosomal dominant disease. The classical paradigm of mutation screening seeks to relate alterations in the exostosin glycosyltransferase genes, EXT1 and EXT2, which are responsible for over 70% of HME cases. However, the pathological significance of the majority of these mutations is often unclear.

**Methods:**

In a Chinese family with HME, EXT1 and EXT2 genes were screened by direct sequencing. The consequence of a detected mutant was predicted by *in silico* analysis and confirmed by mRNA analysis. The EXT1 and EXT2 mRNA and protein levels and the HS patterns in the HME patients were compared with those in healthy controls.

**Results:**

A heterozygous transition (c.743+1G>A) in the EXT2 gene, which co-segregated with the HME phenotype in this family, was identified. The G residue at position +1 in intron 4 of EXT2 was predicted to be a 5′ donor splice site. The mRNA analysis revealed an alternative transcript with a cryptic splice site 5 bp downstream of the wild-type site, which harbored a premature stop codon. However, the predicted truncated protein was not detected by western blot analysis. Decay of the mutant mRNA was shown by clone sequencing and quantification analysis. The corresponding downregulation of the EXT2 mRNA will contribute to the abnormal EXT1/EXT2 ratio and HS pattern that were detected in the patients with HME.

**Conclusion:**

The heterozygous mutation c.743+1G>A in the EXT2 gene causes HME as a result of abnormal splicing, mRNA decay, and the resulting haploinsufficiency of EXT2.

## Introduction

Hereditary multiple exostosis (HME, OMIM 133700 and OMIM 133701) is an autosomal dominant disorder characterized by the presence of cartilage-capped multiple exostoses (osteochondromas) [1]. Osteochondromas usually appear and develop gradually in early childhood and are localized mainly in the juxtaepiphyseal region of long tubular bones. The most serious complication is the malignant transformation of osteochondromas towards chondrosarcomas, which occurs in 1–2% of cases. Penetrance is estimated between 66 and 100% [2]–[4].

HME is a genetically heterogeneous disease that is associated with at least two chromosomal loci: EXT1 (exostosin 1, 8q24.1, OMIM 133700) and EXT2 (exostosin 2, 11p11-p13, OMIM 133701) [5]–[8]. All members of the EXT gene family share a homologous carboxyl terminus and encode glycosyltransferases involved in the biosynthesis of heparin sulfate (HS) [9]. Mutations in the EXT1 and EXT2 genes have been reported to be involved in the pathogenesis of HME and are responsible for about two-thirds and one-third of HME cases, respectively [10], [11].

In this study, we sequenced the exons and the adjacent intronic regions and screened for mutations in the EXT1 and EXT2 genes in a Chinese pedigree with HME. After linkage and mutation analyses, a single-base mutation (c.743+1G>A) was detected in the EXT2 gene. This mutation is located in the donor splice site of intron 4, which was previously thought to be associated with multiple osteochondroma in a sporadic patient, although without sufficient evidence [12]. Here, we showed that this single-base substitution co-segregated within the HME family. Because the consequence of this change was unknown and the molecular basis of the mutation in EXT2 was still unclear, the effect of the potential splice site mutation on RNA processing and its pathogenic mechanism for HME were investigated in the present study.

## Methods

### Ethics Statement

Written informed consent was obtained from all subjects prior to their participation in the experimental protocol. The study was approved by the institutional review board of Nanjing University School of Medicine, Nanjing, China, and consistent with the Declaration of Helsinki.

### Subjects

Blood specimens were collected from 23 members of a Chinese multigeneration pedigree with HME. Nine of the 23 subjects were affected, while other family members and a group of healthy people enrolled in mutation screening. Tissues from osteochondromas of patients in this family were collected and those from patients with osteoarthritis were chosen as controls. Both patients with HME and controls were age/gender matched, and they were males and in their fifties.

### Mutational screening

Genomic DNA of the proband (the first patient in the family to be seen by a doctor) and family members was extracted from peripheral blood samples (TIANamp Blood DNA Kit, Beijing, China). DNA samples were subjected to mutation screening by amplification of segments of the EXT1 and EXT2 genes using primers synthesized based on the intronic sequences of the two genes (EXT1, GenBank accession number NC_000008); EXT2, GenBank accession number NG_007560) (Table 1).

**Table 1:** Primers used in mutation screening of the EXT1 and EXT2 genes.

	Forward primer5′→3′	Backward primer 5′→3′	Product size(bp)
***EXT1 gene***			
E1-1	GTCGTGTCGGGAATGGAA	GACGAAGTGATTGCCTTGC	461
E1-2	CAGGGATTTGTGAGGTTACGG	GCCTGGGTCAAGAGGATTGT	543
E1-3	TGGAGCTGAAAGTGTTGAT	GTTGGCATCTCGCTTCT	344
E1-4	TCGTTCCTTGGGATCAAT	AGTTGGGTCGGAAGTTTT	495
E1-5	CGTGGGGTTTGACATCGG	CCAAGGCTGACTCCCAAA	364
E2	CCAACCTCCTTCCTCAAA	ATCCTCAAGGGAAACCAC	370
E3	GTCGCTTTCCTCACATTC	GAGCTGACCTTTTGGATT	240
E4	CTCTTTGCAGCTGACACTTC	CTCCCCATGTAGGTTTTCCT	519
E5	TACTCTGACTGCCACCAT	GTAAACAAGGGCAACTCC	270
E6	TGTATTTGGACACTGGGTA	AGCAGGGTATGATGTTAGA	556
E7	TGAGAAGAGGCTTTGGGTT	AAACCAAGGCTCCACAGT	222
E8	GAGATTCCTTCGGTGTTG	CAAGGCACGGCTAAAA	325
E9	AGTGGGGAGAAGGTAATG	ATGCCAAGAGGTTTCACT	417
E10	CCTGCCTTGTAGGCTCCTTATG	TGGGTGGAACAGCTAGAGGAA	506
E11-1	GCTCATTTGCCTGACTCC	CACAATCTGGCTCTGCTG	374
E11-2	GAGACATTGAGCGACTTTG	TTCCACGAAGTTTGAGC	476
***EXT2 gene***			
E1	CGTGGTGTCTCGTTTGGGTT	CGAAGGTTGGCTAGGAGAACAG	634
E2	CTGTTCTCCTAGCCAACCTTCG	TCCCACCGAATGTAACAAAGC	598
E3-1	GCAGGCTGATAGATTGAG	GCCACAGCGATAGACAT	640
E3-2	ACCCTCTTCTCCATTGTC	TTCCCTTTAGTTCCCTGA	522
E4	GGGATTTCCAGGAGTTTGC	GGCAAATGCTCCCGAAT	548
E5	CCGAGATGCGTGTATAAGGC	GGACCCTACCCTGTAACTGAT	390
E6	AACTGTTCCCAAATAAGATGTG	CCTGAGCCTTTGCGAGA	480
E7	TGCTTGGCGTCAACCCT	TAAGCCCTGTATGTCATCCAAT	561
E8	AGGAGGTTTGGGATGTTG	TGCTATGGTAAGACCCTAAAA	444
E9	TGTGCCTGGTTGGAGTG	GTCAATGTTCTAAGGGTGGAT	583
E10	CTGTCTCGCTTGCTCACT	GCACTTAGCACAGTCCCT	575
E11	GGAACATCTCCAGAATCCCATT	GCAAGCTGGAAATAGCACCTG	557
E12	GGTCACTTGACCAAAAGCATTC	CAATGTGACCGCATCAATCAT	459
E13	TGGCAGAATAACTAACACCT	AAGGCTCACAATACAATCC	674
E14	ATGCCTTGGCTATGCTGC	ACCGCATCAATCATAGAACCT	360
E15	GACAGAGTTGAATGGAGGAA	GACCTGGGCTTGAACTAAC	403
E16-1	GGGTTAAGAATTGTTGGC	CCCAGCCTCACATTCAG	560
E16-2	CTTCGGGACCATGCCTCT	CAAATGGGCTAACACGCTTC	492
E16-3	AGGAAACTTCACGGACAG	TCAAGGGAGAAAGGTGGTT	375
E16-4	CTGGAGTGCTGGGCTTG	GGGTCACAATTCCCACAAC	465
E16-5	AAGCGTAAGAAGGTCCCA	AGCATAATAACTGCCCACC	631

All the exons and exon–intron boundaries of EXT1 and EXT2 were amplified by polymerase chain reaction (PCR). The products were examined on 2% agarose gel and purified on QIAquick columns (Qiagen Inc, Valencia, CA, USA). This was followed by direct DNA sequencing using an ABI Prism 3100 Genetic Analyzer (Applied Biosystems, Foster City, CA, USA) with both the forward and backward primers.

### In silico prediction analysis

The EXT2 gene sequences from 43 different species were downloaded from NCBI (http://www.ncbi.nlm.nih.gov/). Multiple sequence alignments were performed using ClustalX with standard settings [13].

Bioinformatics analysis of potential splicing aberrations was done using two different web-based programs designed to detect putative splice sites, taking into account branch points, exonic and intronic motifs, and several regulatory proteins. CRYP-SKIP (http://cryp-skip.img.cas.cz/) was used to estimate the probability of cryptic splice-site activation (P_CR-E_) and exon skipping (1-P_CR-E_) as a result of splicing mutations [14]. The CRYP-SKIP algorithm uses a multiple logistic regressions to perform splice site prediction from a mutated sequence. The splice site prediction program at BDGP (http://www.fruitfly.org/about/index.html) was used to predict cryptic splice sites and the influence on splice sites in the mutant sequence [15]. The BDGP algorithm uses a generalized hidden Markov model to recognize donor and acceptor sites.

### Analysis of EXT2 mRNA

Total RNA was isolated from explant-derived cultures from HME patients and controls using Trizol standard procedures. To synthesize cDNA, 5 µg total RNA was reverse transcribed using the random primers (TaKara Biotechnology, Dalian, China) and EasyScript ReverseTranscriptase (TransGen Biotech, Beijing, China). The cDNA products were used directly as templates for PCR amplification. The primers used for the EXT2 human cDNA were: forward: 5′-GGGAGTATAATGAACTGCTCA-3′; reverse: 5′-GCTCCACGAAGAACCACA-3′. The expected sizes of the EXT2 mRNA and cDNA were 3651 and 589 bp respectively. The PCR products were resolved by 1.5% agarose gel electrophoresis and visualized by ethidium bromide staining (Sigma-Aldrich, Beijing, China). The nucleotide sequences were determined by direct sequencing of the PCR products (ABI 3100 DNA sequencer, Applied Biosystems).

The repeated PCR products were cloned into the PMD-18-T vector (TaKaRa Biotechnology, Dalian, China) and amplified in the *E. coli* TOP10 reference strain. Clones were picked up through blue-white spot screening on AMP/IPTG/X-Gal L-agar plates. After plasmid extraction, sequencing of the clones was conducted to evaluate the percentage of alternative transcripts that was present.

### Expression of mRNA and protein

Total RNA was isolated from the exostosis tissue from HME patients and from the juxtaepiphyseal region of bone from the controls, and reverse transcribed into cDNA. All cDNA samples were amplified using SYBR Green PCR Master Mix (Roche Diagnostics GmbH,Mannheim, Germany), and detected using the Step One Real-Time PCR System (Applied Biosystems). Real-time PCR was performed with the primers for EXT1 (forward 5′-GGGGAGAAAATCGCCGAAAGT-3′, reverse 5′- CAT ACTGAGGTGACAACTGGTC-3′) and the primers for EXT2 (forward 5′-GGGATCGAGGTACGAATCACC-3′, reverse 5′-GCCGGTAAGTCCACGTAGAAA-3′). Both the EXT2 primers were located upstream of the mutation site that was detected (c.743+1G). The PCRs were performed in duplicate for each primer/cDNA set with cDNAs from three different transfections. The expression level of the housekeeping gene GAPDH in each sample was used as an internal control, and data analysis was performed using the ΔΔC_T_ method.

Total protein was extracted from the HME patient tissue and control bone samples that are described above. An equal amount of protein from each sample was separated on a SDS-PAGE and western blot. Immunoblot analysis was performed to detect EXT1, EXT2, and HS with the following antibodies: EXT1, rabbit polyclonal antibody H00002131-D01P from Abnova; EXT2, rabbit polyclonal antibody ab102843 from Abcam; and HS, rat monoclonal [A7L6] to HS proteoglycan (large) ab2501, from Abcam. The primary antibody for EXT2 targeted amino acid sequences 188–218 of human EXT2, a region upstream of the mutation. An anti-β-actin antibody was used as an endogenous control for the western blot analysis. Each sample was tested twice in duplicate.

### Statistical analysis

We used an unpaired Student's t-test to evaluate the statistical significance of the experiments. Differences were considered as significant at *P*<0.05.

## Results

### Clinical description of the family with HME

**Figure 1:**
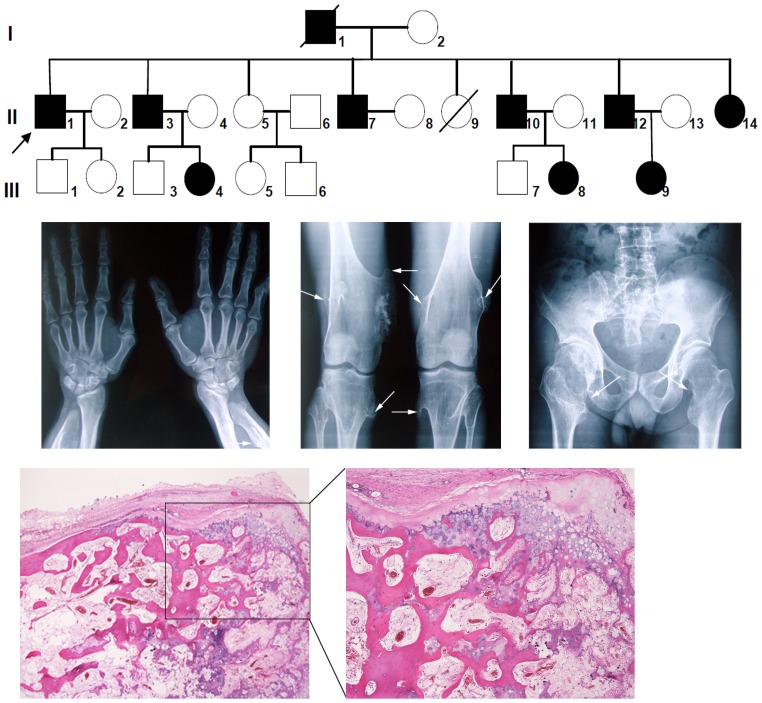
Hereditary and clinical features of a Chinese family with HME. (A) Pedigree of the family with HME. Arrow indicates the proband. Filled circles and squares indicate affected individuals. Open circles and squares indicate normal individuals. Oblique lines indicate the deceased. (B) Postero-anterior positioned radiograph of exostoses (indicated by arrows) in some severely affected individuals. Left image shows the exostosis in juxtaepiphyseal region of right ulna. Middle image shows multiple exostotic lesions in the femur, tibia, and fibular of both lower limbs. Right image depicts excrescences in metaphyseal regions of the femur both sides proximally. (C) Histological lesions of chondrosarcoma in proband under different magnifications (hematoxylin-eosin (HE) stain). One chondrosarcoma site is shown.

According to the anamnesis of some family members, at least ten individuals in the whole family were severely affected with multiple large exostoses. The multigeneration pedigree is shown in Figure 1A. Of the ten known exostosis patients in this family, nine were available for this study. Some of the patients were confirmed by regional X-radiographic analysis to have exostoses and some were also confirmed by pathologists after surgery on the exostoses.

The local appearance and radiological findings in some of the severely affected patients are shown in Figure 1B & 1C. Clinically, all the severely affected members had multiple exostoses at the metaphyses of the long tubular bones, and some of them had 30–40 exostoses all over their bodies. All the lesion sides reported were detected in this one family (Figure 1B). In at least three of the severely affected patients (including the proband), an exostosis had transformed into a chondrosarcoma (Figure 1C).

### Mutation analysis and identification of c.743+1 G>A in EXT2

**Figure 2:**
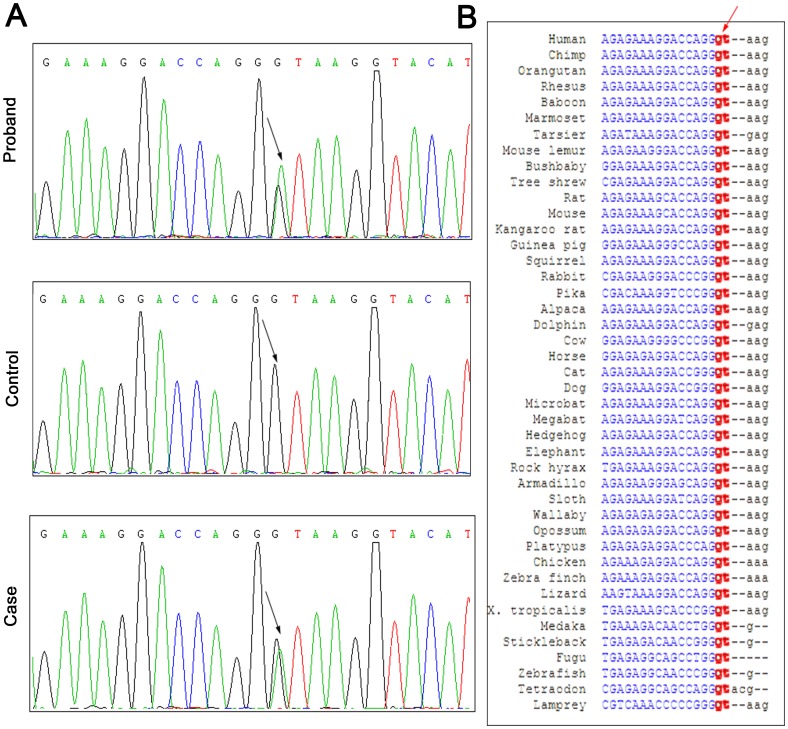
Mutation analysis and identification of EXT2. (A) DNA sequences of the EXT2 gene. Arrows indicate the heterozygous G to A transition site in intron 4 of EXT2 from the proband (upper) and one affected individuals (lower). The mutation was not detected in normal family members or in the healthy controls of the same ethnic origin (control, middle). (B) Alignment of EXT2 gene sequences from 43 species. Conservative character analysis indicated that the G residue (shown in red) at the first position of intron 4 was a highly conserved splicing donor site.

After sequencing all the exons and flanking regions of the EXT1 and EXT2 genes in the proband samples, no mutation was detected in the EXT1 sequences, but a heterozygous point transition (c.743+1G>A, intron 4) was identified in one of the EXT2 sequences (Figure 2A, *upper*). Subsequently, this variation was detected in all the affected individuals in this pedigree by PCR direct sequencing, and was not detected in any of the normal family members or in the healthy controls (Figure 2A, *middle and lower*). This finding suggested that this point transition was co-segregated with HME in this family.

Sequence analysis showed that c.743+1G>A is an intronic variation that is involved in a splicing donor site (GGgt to GGat). DNA sequences from 43 different species were compared to detect variations at this position (Figure 2B). We found that the G residue at this position was highly conserved and that *gt was the splicing donor site in all 43 species. In addition, this G>A variation was reported previously in a sporadic patient with multiple osteochondromas [12]. These findings imply that the c.743+1G>A variation in the EXT2 gene might be the pathogenic splice mutation responsible for the phenotype in this family.

### Aberrant EXT2 splicing transcript

**Figure 3:**
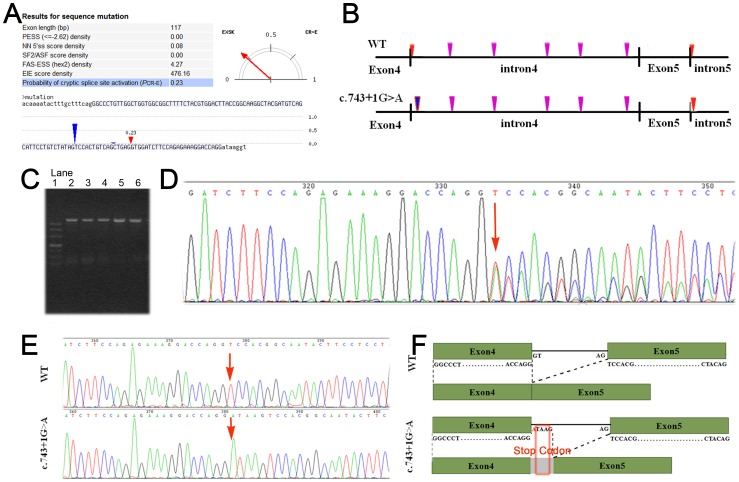
Aberrant EXT2 splicing transcripts with premature termination codon. (A) Screenshot of a CRYP-SKIP output. The input EXT2 sequences included exon 4 (upper) and the flanking intronic sequences (lower). The table on the left lists the summarized values of the predictor variables used in CRYP-SKIP (PESS, putative exonic splicing silencers; NN 5'ss, neural network 5' splice sites; SF2/ASF, the most important SR protein for aberrant splice-site activation, FAS-ESS, ESSs discovered by a fluorescence-activated screen; EIE, exon and intron identity element). PCR-E (shown in light blue) for the mutated sequence is 0.23 in favor of exon skipping. The red vertical mark in the sequence indicates the predicted cryptic donor splice site. The blue vertical mark indicates the predicted acceptor site. (B) BDGP prediction. The red vertical mark indicates the authentic splice sites. The vertical mark indicates the predicted cryptic 5' splice sites with scores >0.90. The blue vertical mark shows the decoy splice site that was confirmed by PCR. (C) Electrophoresis of RT-PCR products derived from the proband, affected individuals, and the controls. Lane 1: DNA marker; Lanes 2, 4, 6: proband and cases; Lanes 3, 5: controls. (D) Direct sequencing of RT-PCR products. Arrow shows the heterozygous insertion of one cryptic splice site 5 bp downstream of the original splice donor site. (E) Clone sequencing of RT-PCR products. The mutant mRNA sequence (c.743+1G>A) has an ATAAG insertion (arrow) compared with the wild-type (WT). (F) Schematic representation of wild-type and aberrant mRNA transcripts. In the wild-type sequence (WT) splicing occurred at the authentic splice sites. In the mutant mRNAs, splicing occurred at the decoy splice site in intron 4 the five additional nucleotides (ATAAG) of intron4 were inserted, which generated the premature termination codon UAA.

Mutations that affect human splice sites can lead to two major phenotypes: exon skipping, and activation of cryptic splice sites close to the authentic splice sites. Our CRYP-SKIP analysis of the mutant EXT2 sequence demonstrated that the c.743+1G>A variation could lead to two possible scenarios: skipping of the fourth exon or generation of a cryptic 5′ donor site in exon 4 (Figure 3A). The probability of exon 4 skipping (1-P_CR-E_) was 0.77, while cryptic splice site activation (P_CR-E_) in exon 4 was 0.23. The BDGP prediction software was then used to predict mutation-induced cryptic splice sites in the flanking sequences and alternation of transcripts (Figure 3B). BDGP evaluated the scores for potential splice sites and for one decoy donor site (AGgt) that had a score of 0.94. The results showed that the c.743+1G>A mutation would lead to the inactivity of the previous splicing donor site (GGgt) and the subsequent activity of several potential donor sites located between the end of exon 4 and the beginning of intron 5.

No difference in the length of the proband and control EXT2 mRNAs was detected by RT-PCR using 1.5% agarose gel electrophoresis (Figure 3C). However, direct sequencing and clone sequencing of the PCR product revealed an altered transcript with one cryptic splice site in intron 4 of the EXT2 gene (Figure 3D & 3E) located 5 bp downstream of the original splice donor site. This splice site corresponds to one of the sites predicted by the BDGP software. This transcript variant harbors a premature termination codon, which was predicted to result in the deletion of amino acids 249–718 in the EXT2 protein sequence (Figure 3F). None of the other activated splice sites predicted by the BDGP software or the splice sites predicted by CRYP-SKIP were detected among the EXT2 mRNA transcripts in vivo.

### Decay of mutated EXT2 mRNA

Transcripts with premature termination codons usually generate deleterious truncated proteins or trigger an RNA surveillance mechanism and nonsense-mediated mRNA decay (NMD) [16], [17]. To find out the possible effect of the alternative transcript, the mRNA and protein variants of EXT2 were further investigated in the patients with the HME phenotype.

**Figure 4:**
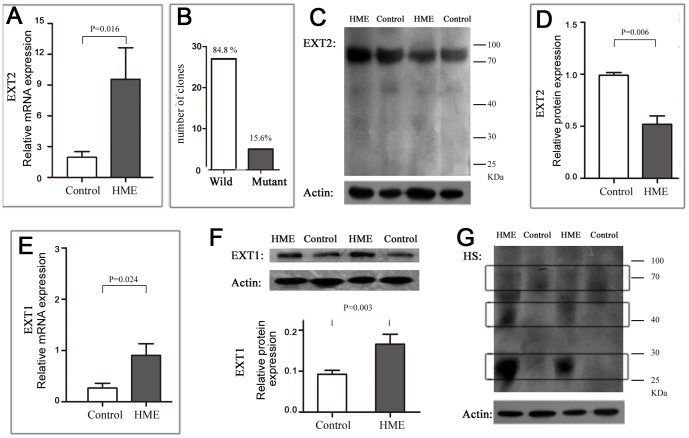
Decay of mutant EXT2 mRNA and protein. (A) Levels of EXT2 mRNA by RT-PCR. The mRNA levels were higher in the patients than that in the controls (*P* = 0.016 in a two-sided Student's t-test). (B) Clone sequencing of wild-type and mutant transcripts. Among the 32 randomly picked clones, 27 (84.4%) were identified as wild-type by direct sequencing, while only five (15.6%) were identified as mutant transcript. (C) Western blots of wild-type and mutant proteins. The band for the predicted truncated protein could not be detected in the HME patients. (D) Comparison of EXT2 protein levels in HME patients and controls. The levels were significantly lower in the patients compared with the controls (*P* = 0.006). (E) Comparison of EXT1 mRNA expression levels in HME patients and controls. Real-time PCR revealed that EXT1 mRNA was more highly expressed in the patients compared with the controls (*P* = 0.024). (F) Comparison of EXT1 protein levels in HME patients and controls. The EXT1 levels higher in the patients compared with the controls (*P* = 0.003). (G) Comparison of HS proteoglycan expression in HME patients and controls. A group of HS proteoglycans around 70 kDa (upper box) were detected in patients and controls; while two HS proteoglycans patterns around 40 kDa and 25–30 kDa (middle and lower boxes) were detected only in the patients.

First, primers that annealed near the 5′-UTR (untranslated region) of the mRNA transcript were constructed. Both primers were upstream of the c.743+1G>A mutation. As shown in Figure 4A, the while levels of EXT2 mRNA in HME patients, containing wild-type and mutant transcripts, were about 4.9 times higher than those in the controls (*P* = 0.016). Clone sequencing revealed an imbalance between the wild-type and mutant transcripts, and the majority of clones (27/32, 84.4%) were wild-type (Figure 4B). This finding indicated that the c.743+1G>A transition leaded to a down-regulation of EXT2 in mutant transcript. Furthermore, the truncated protein, which was predicted to contain the first 248 N-terminal amino acids of EXT2, could not be detected by western blotting (Figure 4C).

In contrast to the mRNA levels, the EXT2 protein levels were significantly lower in the HME patients than in the controls; the ratio of patient to control was about 1/2 (*P* = 0.006; Figure 4C & 4D). In addition, the HME patients had higher EXT1 mRNA and protein levels than the controls (*P* = 0.024 and *P* = 0.003 respectively; Figure 4E & 4F). In the controls, the ratio of the EXT1/EXT2 protein levels was “1”, while in the HME patients the ratio was closer to “3.5”. The HME patients also presented a different pattern of HS proteoglycans than the controls (Figure 4G). In the HME patients, the most abundant HS proteins had molecular weights of around 25–30 kDa and 40 kDa, while in the controls, the most dominant HS proteins were a little less than 70 kDa.

## Discussion

HME is a dominantly inherited skeletal disorder primarily affecting enchondral bone during growth. The disease is characterized by the formation of numerous cartilage-capped benign bone tumors that are often accompanied by skeletal deformities and short stature. In the present study, we found that the HME patients in a Chinese family had almost identical disease manifestations, and some were more severely affected than others and showed the beginnings of chondrosarcoma.

In the studied family, we detected a heterozygous c.743+1 G>A mutation in the EXT2 gene from the HME patients, which might be responsible for the causative defect. The mutation was located in the donor splice site of intron 4 (IVS4+1 G>A) and resulted in an alternative transcript of mRNA with a premature stop codon. The NMD of the mutant mRNA could provoke haploinsufficiency of EXT2, which would then lead to an abnormal EXT1/EXT2 ratio and HS pattern. Our findings illustrate the possible effects of a genetic EXT2 mutation on the HME phenotype, which will help to understand the complex molecular mechanisms underlying the development of exostoses.

The EXT1 and EXT2 genes were identified as tumor suppressor genes for sporadic or inherited exostoses, and many different mutations in these genes have reported and are available in the Multiple Osteochondroma Mutation Database (http://medgen.ua.ac.be/LOVD). At least 710 different EXT1 and 386 different EXT2 mutations have been detected, and there is evidence that mutations in these two genes are responsible for over 70% of the HME cases [11]. Among the 386 EXT2 mutations, there are 267 substitutions, 81 deletions, 19 duplications, eight insertions, eight insertion/deletions, and six are complex mutations. The substitution of one nucleotide in intron 4 of EXT2 (c.743+1 G>A) in a patient with sporadic exostoses, was described previously by Vink et al. [12]. However, in the present study, we have demonstrated for the first time the molecular and cellular basis of the mutation, as well its relation with exostosis formation in HME patients.

The G to A transition at c.743+1 in the EXT2 gene is suggested as a splice site mutation. Increasing evidence has shown that point mutations in the 5′ donor splice site, particularly mutations in the G residue at position +1, are rather common [18]. This notion was confirmed in the present study and the high evolutionary conservation of the G residue was shown across many different species. Each donor splice site variant reduces the pairing of the donor splice site with the complementary site in the small nuclear ribonucleoprotein particle U1snRNP, which is one of the first steps in the complex process of mRNA splicing [19]. Consequently, mutations at donor splice sites can either lead to exon skipping, intron retention, or activation of a cryptic splice site [14], [19], [20].

We used two *in silico* approaches, CRYP-SKIP and BDGP, to predict the effect of c.743+1 G>A on EXT2 splicing and found that the *in silico* analysis of splicing mutations could be a useful aid. By combining the results from different methods, abundant predictive information about mRNA splicing defects can be obtained. However, on bench mRNA analysis is still necessary, especially when there are inconsistencies among the different predictions.

The mRNA analysis indicated that the EXT2 protein encoded by altered transcript had lost 470 amino acids from the C-terminal end and contained only 248 residues instead of the 718 residues in the wild-type protein. Several truncated EXT2s have been predicted to be associated with exostosis and/or HME. Heinritz et al. [21] described a mutant transcript in a patient with sporadic exostosis, which probably encoded for a shortened EXT2 protein of 688 amino acids. Wu et al. [22] predicted that an aberrant mRNA of EXT2 in a patient with HME would produce a truncated protein of 403 amino acids. Xiao et al. [23] predicted that a frameshift and a premature stop codon would lead to the expression of a truncated EXT2 with only 281 amino acids in HME patients. Yang et al. [24] demonstrated that aberrant splicing in the mRNA, causing a shift in the codon-reading frame, terminated with a stop codon at position 404. Together, these findings indicated that the accumulation of mutant proteins might be one of the pathogenic mechanisms of exostosis diseases.

However, the truncated EXT2 protein predicted by mRNA analysis could not be detected in our HME patients. Furthermore, the clone sequencing revealed the downregulation of the altered transcript with the premature stop codon. Using primers located upstream of the mutant site, the sum of the normal and variant mRNA levels was greater in the patients than in the controls. However, the wild-type EXT2 protein level was significantly lower in the patients than in the control, and the mutant EXT2 protein could not be detected.

The mechanisms for this kind of haploinsufficiency have been investigated in many studies and NMD is thought to be one possible explanation [25]–[27]. NMD has been described in several other diseases as a surveillance pathway that prevents the accumulation of truncated proteins and regulates the expression of alternative splice products [16], [17], [28]. The premature termination codon in the alternative EXT2 transcript in our study was situated much further than 50–55 nucleotides upstream of the splicing-generated exon-exon junction and was located more towards the 5′ end than towards the 3′ end of the transcript. Such a situation is likely to elicit NMD and the reduction of transcript levels [29]–[32] leading to haploinsufficiency of EXT2, which would contribute for the exostosis phenotype in patients carrying the c.743+1 G>A mutation.

Although HME has long been linked to molecular defects in the EXT loci, it has only recently been shown that the proteins encoded by these genes are associated with HS biosynthesis. The EXT1 and EXT2 proteins form a hetero-oligomeric complex that is tightly associated with the glycosyltransferase activities involved in the polymerization of HS [9]. Lack of HS leads to focal over-proliferation and adjacent bone collar deficiency and osteoporosis, which can then contribute to osteochondroma pathogenesis [33]. McCormick et al. [34] showed that a missense mutation in EXT1 could leaded to an inactive pool of EXT1/EXT2 complexes. The present study showed that the haploinsufficiency of EXT2 that resulted from the c.743+1 G>A mutation, could lead to an imbalance in the EXT1/EXT2 ratio and abnormal polymerization of HS. Impaired function of HS might be critical in the development of exostoses.

In summary, we discovered a mutation (c.743+1 G>A) in the EXT2 gene that co-segregated with the HME phenotype in a Chinese pedigree. The mutation was shown to be a splicing mutation, which led to an aberrant transcript with a premature termination codon and nonsense-mediated mRNA decay. The decreased expression of EXT2 resulted in an abnormal EXT1/EXT2 ratio and HS pattern and led to the development of exostoses. Already, a number of mutations in the EXT2 gene have been shown to be associated with HME, and more are being reported. However, little information is available about the genotype–phenotype correlation. The present study is the first to report the sequence of the disease-causing mutation, and the molecular mechanism underlying the HME phenotype. Our findings will not only be useful for genetic diagnosis, but will also provide insights for exostosis pathogenesis.

## References

[1] Pannier S, Legeai-Mallet L (2008) Hereditary multiple exostoses and enchondromatosis. Best Practice & Research in Clinical Rheumatology 22: 45–54. 10.1016/j.berh.2007.12.004 18328980

[2] Sugiura Y, Sugiura I, Iwata H (1976) HEREDITARY MULTIPLE EXOSTOSIS - DIAPHYSEAL ACLASIS. Japanese Journal of Human Genetics 21: 149–167. 1088427

[3] Wicklund CL, Pauli RM, Johnston D, Hecht JT (1995) NATURAL-HISTORY STUDY OF HEREDITARY MULTIPLE EXOSTOSES. American Journal of Medical Genetics 55: 43–46. 7702095 10.1002/ajmg.1320550113

[4] Xia CY, Wang J, Zhang SZ, Van Hul W, Wuyts W, et al (2001) A novel deletion mutation of the EXT2 gene in a large Chinese pedigree with hereditary multiple exostosis. British Journal of Cancer 85: 176–181. 11461073 10.1054/bjoc.2001.1880 PMC2364055

[5] Ahn J, Josefludecke H, Lindow S, Horton WA, Lee B, et al (1995) CLONING OF THE PUTATIVE TUMOR-SUPPRESSOR GENE FOR HEREDITARY MULTIPLE EXOSTOSES (EXT1). Nature Genetics 11: 137–143. 7550340 10.1038/ng1095-137

[6] Stickens D, Clines G, Burbee D, Ramos P, Thomas S, et al (1996) The EXT2 multiple exostoses gene defines a family of putative tumour suppressor genes. Nature Genetics 14: 25–32. 8782816 10.1038/ng0996-25

[7] Wuyts W, VanHul W, Wauters J, Nemtsova M, Reyniers E, et al (1996) Positional cloning of a gene involved in hereditary multiple exostoses. Human Molecular Genetics 5: 1547–1557. 8894688 10.1093/hmg/5.10.1547

[8] Li YC, Wang DB, Wang WB, Wang J, Li HY, et al (2009) Identification of Four Novel EXT1 and EXT2 Mutations in Five Chinese Pedigrees with Hereditary Multiple Exostoses. Genetic Testing and Molecular Biomarkers 13: 825–830. 19839753 10.1089/gtmb.2009.0083

[9] Senay C, Lind T, Muguruma K, Tone Y, Kitagawa H, et al (2000) The EXT1/EXT2 tumor suppressors: catalytic activities and role in heparan sulfate biosynthesis. Embo Reports 1: 282–286. 11256613 10.1093/embo-reports/kvd045 PMC1083719

[10] Bovee JV (2008) Multiple osteochondromas. Orphanet Journal of Rare Diseases 3.. 10.1186/1750-1172-3-3 PMC2276198 18271966

[11] Jennes I, Pedrini E, Zuntini M, Mordenti M, Balkassmi S, et al (2009) Multiple Osteochondromas: Mutation Update and Description of the Multiple Osteochondromas Mutation Database (MOdb). Human Mutation 30: 1620–1627. 19810120 10.1002/humu.21123

[12] Vink GR, White SJ, Gabelic S, Hogendoorn PCW, Breuning MH, et al (2005) Mutation screening of EXT1 and EXT2 by direct sequence analysis and MLPA in patients with multiple osteochondromas: splice site mutations and exonic deletions account for more than half of the mutations. European Journal of Human Genetics 13: 470–474. 15586175 10.1038/sj.ejhg.5201343

[13] Thompson JD, Gibson TJ, Plewniak F, Jeanmougin F, Higgins DG (1997) The CLUSTAL_X windows interface: flexible strategies for multiple sequence alignment aided by quality analysis tools. Nucleic Acids Research 25: 4876–4882. 9396791 10.1093/nar/25.24.4876 PMC147148

[14] Divina P, Kvitkovicova A, Buratti E, Vorechovsky I (2009) Ab initio prediction of mutation-induced cryptic splice-site activation and exon skipping. European Journal of Human Genetics 17: 759–765. 19142208 10.1038/ejhg.2008.257 PMC2947103

[15] Reese MG, Eeckman FH, Kulp D, Haussler D (1997) Improved splice site detection in Genie. Journal of Computational Biology 4: 311–323. 9278062 10.1089/cmb.1997.4.311

[16] Gerards M, van den Bosch B, Calis C, Schoonderwoerd K, van Engelen K, et al (2010) Nonsense mutations in CABC1/ADCK3 cause progressive cerebellar ataxia and atrophy. Mitochondrion 10: 510–515. 20580948 10.1016/j.mito.2010.05.008

[17] Inoue K, Khajavi M, Ohyama T, Hirabayashi S, Wilson J, et al (2004) Molecular mechanism for distinct neurological phenotypes conveyed by allelic truncating mutations. Nature Genetics 36: 361–369. 15004559 10.1038/ng1322

[18] Houdayer C, Dehainault C, Mattler C, Michaux D, Caux-Moncoutier V, et al (2008) Evaluation of in silico splice tools for decision-making in molecular diagnosis. Human Mutation 29: 975–982. 18449911 10.1002/humu.20765

[19] Matlin AJ, Clark F, Smith CWJ (2005) Understanding alternative splicing: Towards a cellular code. Nature Reviews Molecular Cell Biology 6: 386–398. 15956978 10.1038/nrm1645

[20] Sakabe NJ, de Souza SJ (2007) Sequence features responsible for intron retention in human. Bmc Genomics 8.. 10.1186/1471-2164-8-59 PMC1831480 17324281

[21] Heinritz W, Huffmeier U, Strenge S, Miterski B, Zweier C, et al (2009) New Mutations of EXT1 and EXT2 Genes in German Patients with Multiple Osteochondromas. Annals of Human Genetics 73: 283–291. 19344451 10.1111/j.1469-1809.2009.00508.x

[22] Wu YH, Xing XS, Xu SN, Ma HW, Cao LH, et al (2013) Novel and recurrent mutations in the EXT1 and EXT2 genes in Chinese kindreds with multiple osteochondromas. Journal of Orthopaedic Research 31: 1492–1499. 23629877 10.1002/jor.22378

[23] Xiao CY, Wang J, Zhang SZ, Van Hul W, Wuyts W, et al (2001) A novel deletion mutation of the EXT2 gene in a large Chinese pedigree with hereditary multiple exostosis. British Journal of Cancer 85: 176–181. 11461073 10.1054/bjoc.2001.1880 PMC2364055

[24] Yang L, Hui WS, Chan WCW, Ng VCW, Yam THY, et al (2010) A Splice-Site Mutation Leads to Haploinsufficiency of EXT2 mRNA for a Dominant Trait in a Large Family with Multiple Osteochondromas. Journal of Orthopaedic Research 28: 1522–1530. 20872591 10.1002/jor.21162

[25] Bateman JF, Freddi S, Nattrass G, Savarirayan R (2003) Tissue-specific RNA surveillance? Nonsense-mediated mRNA decay causes collagen X haploinsufficiency in Schmid metaphyseal chondrodysplasia cartilage. Human Molecular Genetics 12: 217–225. 12554676 10.1093/hmg/ddg054

[26] Fang P, Schwartz ID, Johnson BD, Derr MA, Roberts CT Jr, et al (2009) Familial Short Stature Caused by Haploinsufficiency of the Insulin-Like Growth Factor I Receptor due to Nonsense-Mediated Messenger Ribonucleic Acid Decay. Journal of Clinical Endocrinology & Metabolism 94: 1740–1747. 19240156 10.1210/jc.2008-1903

[27] Frio TR, Wade NM, Ransijn A, Berson EL, Beckmann JS, et al (2008) Premature termination codons in PRPF31 cause retinitis pigmentosa via haploinsufficiency due to nonsense-mediated mRNA decay. Journal of Clinical Investigation 118: 1519–1531. 18317597 10.1172/JCI34211 PMC2262031

[28] Baserga SJ, Benz EJ (1988) NONSENSE MUTATIONS IN THE HUMAN BETA-GLOBIN GENE AFFECT MESSENGER-RNA METABOLISM. Proceedings of the National Academy of Sciences of the United States of America 85: 2056–2060. 3353367 10.1073/pnas.85.7.2056 PMC279927

[29] Carter MS, Li SL, Wilkinson MF (1996) A splicing-dependent regulatory mechanism that detects translation signals. Embo Journal 15: 5965–5975. 8918474 PMC452383

[30] Maquat LE (2004) Nonsense-mediated mRNA decay: Splicing, translation and mRNP dynamics. Nature Reviews Molecular Cell Biology 5: 89–99. 15040442 10.1038/nrm1310

[31] Nagy E, Maquat LE (1998) A rule for termination-codon position within intron-containing genes: when nonsense affects RNA abundance. Trends in Biochemical Sciences 23: 198–199. 9644970 10.1016/s0968-0004(98)01208-0

[32] Wang J, Gudikote JP, Olivas OR, Wilkinson MF (2002) Boundary-independent polar nonsense-mediated decay. Embo Reports 3: 274–279. 11850396 10.1093/embo-reports/kvf036 PMC1084009

[33] McCormick C, Duncan G, Tufaro F (2000) Herpes simplex virus: discovering the link between heparan sulphate and hereditary bone tumours. Reviews in Medical Virology 10: 373–384. 11114076 10.1002/1099-1654(200011/12)10:6<373::aid-rmv291>3.0.co;2-n

[34] McCormick C, Duncan G, Goutsos KT, Tufaro F (2000) The putative tumor suppressors EXT1 and EXT2 form a stable complex that accumulates in the Golgi apparatus and catalyzes the synthesis of heparan sulfate. Proceedings of the National Academy of Sciences of the United States of America 97: 668–673. 10639137 10.1073/pnas.97.2.668 PMC15388

